# Evaluating the impact of Brexit on the pharmaceutical industry

**DOI:** 10.1186/s40545-017-0120-z

**Published:** 2017-10-04

**Authors:** Fawz Kazzazi, Cleo Pollard, Paul Tern, Alejandro Ayuso-Garcia, Jack Gillespie, Inesa Thomsen

**Affiliations:** 1 University of Cambridge, School of Clinical Medicine, Cambridge, UK; 2 King’s College London, London, UK; 3 University of Cambridge, Cambridge, UK; 4 Imperial College London, MRC London Institute of Medical Sciences, London, UK; 5 Polygeia (Global Health Student Think-Tank), Cambridge, UK; 6 Leckhampton House, 37 Grange Road, Cambridge, CB3 9BJ UK

**Keywords:** Brexit, Pharma, Pharmaceutical, Industry, Impact of Brexit, Leaving EU, Drug manufacture, Employment, Workforce, Funding

## Abstract

**Introduction:**

The UK Pharmaceutical Industry is arguably one of the most important industries to consider in the negotiations following the Brexit vote. Providing tens of thousands of jobs and billions in tax revenue and research investment, the importance of this industry cannot be understated. At stake is the global leadership in the sector, which produces some of the field’s most influential basic science and translation work. However, interruptions and losses may occur at multiple levels, affecting patients, researchers, universities, companies and government.

**Goals:**

By understanding the current state of pharmaceutical sector, the potential effect of leaving the European Union (EU) on this successful industry can be better understood. This paper aims to address the priorities for negotiations by collating the analyses of professionals in the field, leading companies and non-EU member states.

**Research methods:**

A government healthcare policy advisor and Chief Science Officer (CSO) for a major pharmaceutical firm were consulted to scope the paper. In these discussions, five key areas were identified: contribution, legislative processes, regulatory processes, research and outcomes, commercial risk. Multiple search engines were utilised for selecting relevant material, predominantly PubMed and Google Scholar. To supplement this information, Government documents were located using the “GOV.UK” publications tool, and interviews and commentaries were found through the Google News search function.

**Conclusion:**

With thorough investigation of the literature, we propose four foundations in the advancement of negotiations. These prioritise: negotiation of ‘associated country’ status, bilaterally favourable trade agreements, minimal interruption to regulatory bodies and special protection for the movement of workforce in the life sciences industry.

## Background

A glance at the stock market suggests that the UK’s pharmaceutical sector has emerged largely unscathed from Brexit, performing comparatively stronger than other industries in the immediate economic uncertainty that followed the referendum result in June 2016. As industries such as banking and insurance grappled with the pound falling to its lowest level in thirty years [1], the pharmaceuticals sector appeared to buoy calmly above the volatility. The British pharmaceutical company, GlaxoSmithKline (GSK), headquartered in Brentford, UK, even saw its share price rise in the immediate aftermath of the vote, highlighting the robustness of the industry [2]. These results panned out promisingly, flouting widespread speculation that the sector would be one of the worst hit. Some in the industry, whilst acknowledging the potential negative impacts of Brexit, even hailed independence from the EU as an opportunity for the UK to leverage its life science sector [3]. Such short-term observations would make an optimistic evaluation of the impact on the industry a seemingly straightforward one to write. However, it would likely prove short-sighted. As negotiations for a post-Brexit world take shape, the UK’s pharmaceutical industry, one of the country’s most reputable sectors, has perhaps more at stake than any other industry owing to the complex nature of its current regulatory, funding and research structures.

The gravity of the potential disruption to the industry is reflected in the fact that the UK government has outlined science and innovation as one of the 12 ‘negotiating priorities’ of Brexit [4]. This is matched by the insistence of industry leaders that a solution be reached swiftly in order to prevent financial damage to the sector and possible risks to all those who depend on the research, products and services it delivers. For example, Steve Bates, BioIndustry Association CEO, has called for an early agreement on issues such as regulation of medicines and the ability of non-UK nationals to work in the UK life science ecosystem, whilst the European Federation of Pharmaceutical Industries and Associations has warned that “disruption could lead to delays in medicines reaching patients” [5].

The pharmaceutical industry is being afforded attention and a sense of immediacy in these early stages of negotiation, yet the details that will determine its future remain unclear. This report aims to inform on the possible options available to the UK pharmaceutical sector now that its relationship with the EU faces potentially drastic changes. It is impossible to predict whether this new affiliation will be one of continuing partnership, lukewarm cohabitation or absolute divorce in terms of the deals reached on regulation, clinical trials, and the movement of persons and drugs (amongst other factors). It is possible, however, to shed light on the intricacies of any one these options, drawing knowledge from the EU’s current relationships with non-EU states. Combining this insight with an outlining of the current state of the UK pharmaceutical sector should provide clearer understanding of where the priorities lie for pharma in these crucial Brexit negotiations.

## Methodology

The impact of Brexit on the pharmaceutical industry is a diverse subject that is placed at the conjunction of economics, politics and science. In order to adequately represent the depth of discussions, the study consulted experts for their guidance in scoping this project. Three experts were selected for their breadth of knowledge: a government public health consultant, a member of parliament (MP) and a Chief Science Officer (CSO) of a major pharmaceutical firm. Following this scoping phase, five key areas were identified for exploration:

- Contribution*

- Legislative processes

 ○ Consideration of post-Brexit models*

  ■ Swiss

  ■ Canadian

  ■ European Economic Area

 ○ Potential cost burden from additional regulatory and market entrance requirements

- Regulatory processes

 ○ European Medicines Agency*

 ○ Medicines and Healthcare Regulations Agency*

 ○ Movement of people*

 ○ Professional standards

 ○ Clinical Trials Directive and Clinical Trials framework*

 ○ The Customs Union

- Research and outcomes*

 ○ Horizon 2020

 ○ Other EU funded projects

 ○ Continued access to EU funding in science and technology

- Creation of reputational and commercial risk for pharmaceutical companies wishing to do business from within and outside the UK

To find relevant literature, composite and extended terms containing the roots “pharm*” and “drug*” were searched with terms relating to Brexit, such as “Brexit”, “EU”, “eur*” and “leave EU”, in search engines Pubmed and Google Scholar. Additionally, the same terms were used to locate government documents via the “GOV.UK” publication search tool. Furthermore, reports and commentaries were found through regulatory body websites and pharmaceutical associations such as “European Medicines Agency”, “Association of British Pharmaceuticals” and “UK Biotech Association”. Articles and interviews were discovered through the use of internet search engines such as “Google News”. Finally, specific numerical figures and anecdotes from notable individuals were sought directly using the aforementioned search tools.

**Fig. 1:**
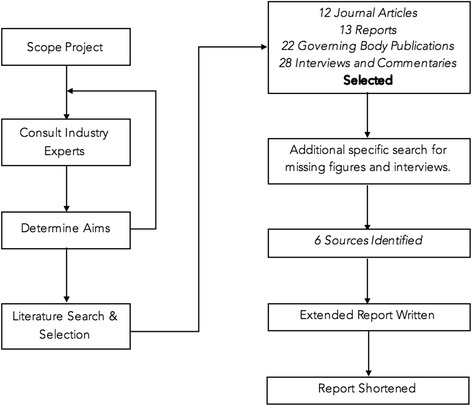
Research framework

The research framework is outlined in Fig. 1. The initial search found 252 documents, of which 79 were used to inform an extended report and 60 of those used for this manuscript (Fig. 1). The items labelled with an asterisk (*) were the focus of this manuscript. Limitations in available literature excluded topics relating to: customs union, future trade risk and new British professional standards.

### Pre-Brexit figures

The pharmaceutical industry constitutes an important component of the UK economy. The UK life sciences sector contributed £30.4 billion in UK GDP, supported 482,000 jobs and contributed £8.6 billion in taxes in 2015 [6], a significant portion (over half) due to the pharmaceutical industry [7]. Two of the world’s largest pharmaceutical companies, AstraZeneca and GSK, are headquartered in the UK and almost all notable multinational pharmaceutical companies maintain a presence in the country.

The UK’s life sciences industry is viewed as one of the most dynamic in Europe and has received substantial foreign investment over the last ten years [8]. Multiple facets to the industry have allowed the UK to become a world leader in scientific research ahead of both China and the US, a feat which has ultimately benefitted the UK [9]. Investors appreciate the fairness and transparency of the UK’s regulatory environment and have benefited from a collaborative government-industry relationship.

#### Industry overview

The pharmaceutical sector employs approximately 70,000 people in the UK [10] and provides jobs in a number of areas: manufacturing, distribution, clinical trials and R&D.

Pharmaceutical manufacturing is one of the few components of the UK’s manufacturing sector to have experienced fairly consistent growth in output, productivity and employment over the last decade. Looking ahead, growth rates of 4–10% per annum had been forecast for the sector [11]. It is the most research intensive component of the UK economy and is responsible for around 25% of all commercial R&D conducted in the UK [12].

**Fig. 2:**
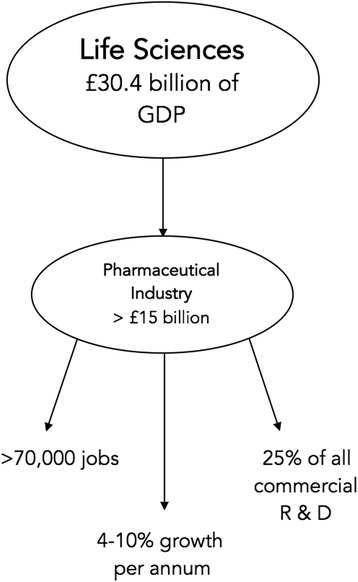
Pharmaceutical Industry at a Glance

The UK is the main location in Europe for venture financing of pharmaceutical companies, accounting for over a third of the total Venture Capital (VC) raised in the pharmaceutical sector in Europe [13]. The London Stock Exchange, including its smaller sub-market, Alternative Investment Market (AIM), is an important source of funding for pharmaceutical companies, although it is not dominant within Europe [14] (Fig. 2).

#### Pharmaceutical manufacturing

The UK’s reliable legal system and strong protection of intellectual property has helped to establish the country as a major centre for the manufacture of medical devices and pharmaceuticals. It is estimated that there are over 500 pharmaceutical manufacturers in the UK [15].

The UK’s domestic market for pharmaceutical products is currently valued at ~£30 billion and demand for pharmaceutical products is expected to grow substantially due to the pressures of an ageing population [16]. Weak economic growth could reduce growth projections for the sector but, in general, demand for healthcare products has been resilient to economic downturns with the sector’s growth remaining positive even during the 2008–09 crisis.

The EU remains the largest single export market for UK pharmaceutical companies. Exports to the EU have grown by around 30% over the last 10 years and further growth is expected. Germany is a crucial market due to its large and wealthy yet rapidly ageing population [15]. However, the EU now represents less than half of total UK pharmaceutical exports. Exports to outside the EU more than doubled over the last ten years. Key growth markets are Asia (especially China) and the US [15].

Drug pricing and reimbursement is an exclusive competency of EU member states. Consequently, third parties can purchase branded pharmaceuticals in EEA member states with lower prices and then resell them in other EU member states [17]. This process is known as parallel importation. Parallel imports of pharmaceutical products were prohibited in Sweden until it joined the EU in 1995; evidence suggests that, since then, parallel imports have reduced pharmaceutical prices [18].

Biosimilar drugs are non-branded near-equivalents of branded biopharmaceutical products. It is possible that the government will also seek to encourage the use of biosimilars over the same period, although these drugs do not offer the same cost savings as generic drugs. The UK government has been focusing on cost reduction measures in recent years and this has included emphasising the use of generic drugs. Spending on generic drugs as a portion of total healthcare spending is expected to rise over the next decade [19].

#### Clinical trials

The UK’s National Institute for Healthcare Research (NIHR) is the largest funder of clinical trial research in the EU [20]. Clinical trials provide important information for academics and R&D departments. The UK’s status as a major location for clinical trials enhances its desirability as a location for pharmaceutical development.

Since 2004, the UK has been party to the EU Clinical Trials Directive (CTD), 2001/20/EC EUCTD, which has received criticism for adding red tape, whilst bringing few tangible benefits and perhaps encouraging clinical trials to take place outside the EU. Sir Michael Rawlins, current chair of the Medicine and Healthcare Products Regulatory Agency (MHRA), referred to the original CTD as a “catastrophe” [21]. Nonetheless, with substantial changes to this directive due to be implemented in 2018, there is little support amongst the research community for leaving the EU-wide clinical trials network.

One key issue is the increased emphasis on rare diseases and genetic research. Both occur highly infrequently, making it impossible to generate a sufficiently large sample in any particular EU country [22]. This necessitates international longitudinal studies and it is feared that the UK will be unable to participate in such studies once outside the framework of the European CTD. That said, the UK is home to “The 100,000 Genomes Project”, a national initiative aiming to sequence the DNA of 100,000 people. This is the largest project of its kind in the world [23].

### Effect of Brexit on the pharmaceutical industry – Post-Brexit

#### Innovation

The pharmaceutical industry is one of the UK’s main motors for innovation. Investing more in R&D than any other sector in the UK (£4 billion in 2014 [24]), the life sciences sector stimulates the creation of highly skilled jobs and the formation of partnerships and collaborations with academia and other sectors, which generates value for the UK.

The UK is a reference internationally in the life sciences industry, having discovered and developed 25 of the top 100 prescription medicines globally [15]. Nevertheless, to sustain the status of global leadership in the sector, it is essential to guarantee long-term funding, the brightest talent and the ability to collaborate at scale. Commercialisation of this research will require funding of small and medium enterprises (SMEs), from inception to sale, or Initial Public Offering (IPO).

The commercialisation and growth of SMEs rely heavily on the UK’s VC, whilst also depending greatly on the European Investment Bank (EIB) and the European Investment Fund (EIF) funding; these constitute 25–40% of VC funds and attract further private investment [25]. If the European Investment Bank (EIB) funding pipeline is broken, UK SMEs will suffer and fewer start-ups will be created.

#### Diminished innovation

Framework Programmes (FPs) are the main EU funding mechanism for research, development and innovation, accounting for 78% of EU research funding received by the UK between 2007 and 2013 (FP7) [26] or 3% of UK’s expenditure on R&D over the same period [27]. As a result of FPs and structural funds for research and innovation activities, the UK secured €8.8 billion in funding from the EU between 2007 and 2013 [28], earning €3.4 billion more than contributed [29].

Horizon 2020 is the current FP with a budget of €74.8 billion available for the period 2014 to 2020 [29]. This amount is distributed based on criteria of scientific excellence, alignment with a number of strategic objectives (‘grand challenges’), geographical and disciplinary diversity, and potential for commercialisation.

The HM Treasury has committed to underwrite funding for approved Horizon 2020 projects applied for before the UK leaves the EU [30], providing short-term reassurance to applicants from the UK’s research and innovation base. Access to EU funding beyond Horizon 2020 is still unknown, which is particularly worrying in the Life Sciences sector where projects can require extended periods of time. However, an individual of any country maintains the right to apply for funding from the European Research Council and the Marie Skłodowska-Curie funding.

#### Loss of global research leader status

Although 19% of the world’s most cited life science academic publications in 2012 were produced by the UK [24], 60% of all internationally co-authored papers are with EU partners [31]. Cross-border collaborations between EU member states are becoming increasingly paramount in achieving the scale required to make breakthrough discoveries. Loss of EU membership presents a considerable obstacle in maintaining the UK at the forefront of global research. Furthermore, if non-EU countries see European scale as indispensable to meeting their objectives, it is likely that they will target partnerships outside of the UK.

Additionally, loss of alignment with the EU on data protection could further endanger the UK’s leading position since the current UK Data Protection Act is insufficient to enable pan-European data sharing.

#### Falling R&D spending

There is a positive correlation between government spending on medical research and private R&D spending, a 1% increase in the former being associated with a 0.7% increase in the latter [32]. Any reductions in public funding could result in a decline in private R&D spending from pharmaceutical companies who, in 2014, spent 16% of their European R&D budget in the UK [33].

The benefit of increased government expenditure on research quality is demonstrated through Singapore’s Agency for Science, Technology and Research (A*STAR), which was established in 1991. This body is credited with improving Singapore’s output to the biotechnology sector by attracting top researchers from around the globe. Its success is believed to be rooted in the lack of stringent regime and control of research targets; investing in the best researchers, not just the best research proposals, has led to an influx of researcher applications [34]. In 2016, it committed 19 billion Singaporean Dollars (~£11 billion) to fund R&D until 2020 [35].

#### Regulation

It is difficult to assess the extent to which the UK’s pharmaceutical industry will continue to be regulated by EU laws once the UK leaves the EU. A large part of this depends on whether the UK will continue to be part of the European single market and support free movement of medicinal products, a decision for both the UK and remaining EU member states to reach. The most likely outcome is that companies seeking to launch new products will have to apply separately for regulatory approval in the UK and in the EU. This will introduce delays to the system and may be detrimental to drug launches in the UK, as companies may prioritise applying for regulatory approval in the considerably larger EU market. As Japan’s Ministry of Foreign Affairs states, the “appeal of London as an environment for the development of pharmaceuticals would be lost” if the EMA relocates, which would in turn drive negative impacts on R&D [36]. Not committing to the full implementation of the European Falsified Medicines Directive (FMD) would deprive the UK of the EU’s efforts to prevent falsified medicines entering EU countries and thus reaching UK patients.

Furthermore, whilst the MHRA has released a statement announcing that it currently remains committed to playing a full and active role in European regulatory procedures for medicines and devices, its position beyond this interim period is not known. Rawlins has expressed the MHRA’s preference for working closely with the EMA and maintaining the current regulatory system to the extent of even contributing to the deliberations of the Scientific Advisory Committee. Ultimately, however, the extent to which the MHRA will continue to engage with the EMA will be determined by Parliament’s Scientific Advisory Body [37]. Regardless of the UK’s path in terms of EU market access, there will be an increased authorisation burden for the UK, as drugs that have already been centrally approved by the EMA would need additional authorisation in the UK.

The EMA has already forecast potentially significant disruptions to its operations following Brexit but it remains unclear as to whether a relocation will take place or what other changes will emerge in terms of the UK’s relationship with the EMA [38].

However, these problems could be circumvented by various administrative streamlining measures such as those used by EFTA states. For example, Liechtenstein uses processes that automatically approve medicines authorised by the EMA, whilst Norway and Iceland remain under the EMA’s umbrella.

In April 2014, a new Clinical Trials Regulation (CTR), Regulation EU No. 536/2014, was adopted by the EU with the aim of full implementation by 2018 [25]. This CTR focuses on the simplification of current rules, streamlining applications for the conduction of clinical trials and their authorisation, and aiming to increase the transparency of the data produced [39]. Should the UK not adhere to Regulation EU No. 536/2014, innovation could be hindered as opportunities for doctors and academics to conduct clinical trials will be restricted and companies will begin to look elsewhere to carry out theirs.

#### Regulation of medical devices

Medical devices are regulated by the EMA and the MHRA. The Medical Devices Directive (MDD) similarly attempts to apply EU-wide standards to medical devices. This means that, at present, devices licensed in one EU country can be sold throughout the EU. This ‘lowest common denominator’ system allows manufacturers to deliberately register their products in countries with lower standards.

With Brexit, the MHRA is likely to impose tighter standards on medical devices, putting in place regulations that the EMA failed to install due to resistance from member states. This will benefit larger pharmaceutical companies with more sophisticated R&D and manufacturing infrastructure for ensuring products are of a high quality. Simultaneously, these regulations may create barriers to entry for new start-ups lacking the capital to produce high quality products to meet the more stringent regulations.

An end to cooperation with the EU on matters of European pharmacovigilance (PV) and future medical device databases (EUDAMED) will diminish the ability of the UK to detect side effects and respond to safety issues. In addition, loss of access to the European Centre for Disease Prevention and Control (ECDC) could hinder the UK’s ability to produce medicines that fight pandemics, and may delay the manufacture and supply of vaccines.

#### Loss of certainty and scale

The Association of the British Pharmaceutical Industry (ABPI) supports.

the current regulatory system, which is regarded as highly effective, but has expressed concern about the potential additional bureaucracy that a new independent UK regulatory system would create [25].

If separate regulatory processes exist for the UK, companies seeking to launch new products will have to apply for regulatory approval in the UK and EU regions, which would cause delays. This could be detrimental to drug launches in the UK, as companies are likely to prioritize applying for regulatory approval in the considerably larger (500 million) EU market; the UK only constitutes 3% of the world’s market for new medicines (60 million). As Rawlins stated: “One of the biggest worries I have about Brexit and standing alone as a regulator is that we are only 3% of the world market for new drugs and, if we are not careful, we are going to be at the back of the queue” [37]. David Jeffreys, spokesperson for the Association of British Pharmaceutical Industries and Vice-President of Eisai, a Japanese pharmaceutical firm, says, “The early innovative medicines will be applied for in the USA, in Japan and through the European system and the UK will be in the second, or indeed the third, wave - so UK patients may be getting medicines, 12, 18, 24 months later than they would if we remained in the European system.” [40].

Conversely, some scientists take a more positive view, arguing that Brexit provides an opportunity for more liberal regulatory rules that will permit drugs to be launched more quickly in the UK [41]. Rawlins has also suggested the possibility of launching a system giving provisional licenses to new medicines whilst more real-world data is being collected, which would make the UK market more attractive for pharmaceutical companies.

#### Influence

The MHRA has a wide range of international links and is respected worldwide as one of the leading regulatory authorities for medicines and medical devices. The MHRA has shared its regulatory expertise with Malta, Latvia and the Czech Republic in a bid to help countries that have recently joined the EU to develop the systems necessary to playing an active part in European regulation [42]. The MHRA was:

- lead regulator in granting licensing to 7 out of 10 European medical products in 2007 [43];

- a rapporteur in 15% of the procedures of the PV Risk Assessment Committee (PRAC) and the Committee for Medicinal Products for Human Use (CHMP) in 2015 [25];

- responsible for inspections that resulted in 25% of Good Manufacturing Practice (GMP) certificates issued in 2015 for sites outside the EU [25].

The UK’s VMD has also played a notable role in regulation, acting as a Reference Member State in 43% of Mutual Recognition Procedures in 2015 [25]. The loss of influence in the European system could deter regulatory experts from living and working in the UK, and result in the future implementation of regulations that are less favourable to UK interests, damage that will worsen if the EMA relocates.

### Talent

#### Leadership

Approximately 17% of Science, Technology, Engineering and Mathematics (STEM) academics in UK research institutions are non-UK EU nationals [44]. Facilitating movement across borders is essential to ensuring the supply of talent demanded in current and emerging skill gap areas such as bioinformatics, genomics or Advanced Therapy Medicinal Product (ATMP) manufacturing.

The UK’s global reference status therefore depends on removing any barriers to attracting, developing and retaining talent. This includes the current state of uncertainty regarding the UK’s future immigration policy and the unwelcoming image projected on foreign workers.

The government remains committed to ensuring researcher mobility is protected. The House of Lords concluded that researcher mobility was “of critical importance to the UK science community, including academia, business and charities” and that “researcher mobility must be protected if UK science and research is to remain world-leading” [45]. A parliament report on the implications and outcomes for science and research concluded by saying: “We understand that the Government is not yet able to offer firmer guarantees regarding future immigration rules for researchers but remind them that this is essential in order to continue to attract top-quality researchers to the UK… There is clear agreement that researcher mobility is a crucial component of the UK’s successful research and science sector.” [46].

#### Headquarters

London is home to the EMA, as well as the European headquarters of over a dozen global pharmaceutical companies, the global headquarters of GSK and AstraZeneca, and considerable R&D and manufacturing operations for Amgen and Pfizer. This has attracted and nurtured talent across the value chain in areas such as research, development, regulation, manufacturing and commerce. GSK and AstraZeneca, for example, will employ 15 and 50 university graduates respectively in 2017 [47, 48]. Outside of the EU, the UK may see its capacity to attract talent significantly reduced, which could result in the relocation of operations, causing losses in job, economic contributions and innovation capacity.

### Consideration of post-Brexit models

#### Initial overview

**Fig. 3:**
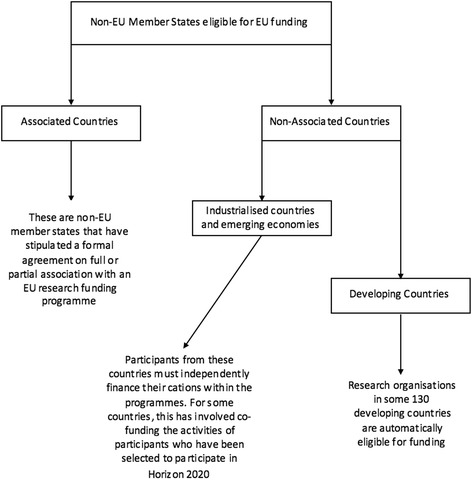
Non-EU member state funding model

There are three existing models that could provide a solution which would allow the UK to continue receiving EU funding and benefitting from its association with EU-driven scientific research actions (Fig. 3).

A further, and likely, route will be that the UK negotiates its own model with the EU as it seeks to protect its current and future research funding. It should be noted that, even if the UK were able to adopt an existing model, such as that of an ‘associated country’, additional negotiations will be inevitable [49].

#### Associated countries

These are non-EU member states that have stipulated an individual formal agreement on full or partial association with an EU research funding programme. To be involved in these programmes in the same manner as EU member states, these countries must pay a fee which is calculated based on their GDP and on further negotiations.

Nevertheless, whilst these countries can receive and benefit from EU research funding, they cannot influence the direction of these programmes as access does not grant them a voice in the European Council or European Parliament. This is the key difference between EU member states and ‘associated countries’.

Since the referendum result, lobbying by Universities UK (UUK) has sought to put pressure on the UK government to push negotiations for ‘associated country’ status [49]. This would secure the UK’s participation in Horizon 2020 in a similar manner to other ‘associated countries’ [49].

#### Non-associated third countries

These are non-EU member states, such as Afghanistan and Argentina, which are not formally associated with EU research funding programmes and considered as ‘developing’ or ‘industrialised’. Nevertheless, organisations and participants from these countries can become partners with the programmes and receive funding.

#### The pharmaceutical industry

**Fig. 4:**
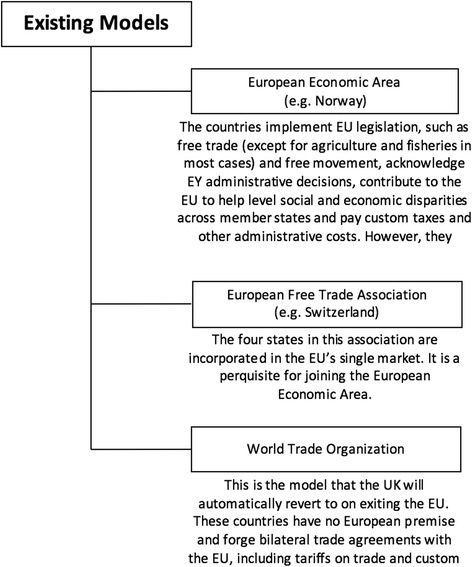
Existing trade models in the EU

In considering the post-Brexit options for the UK pharmaceutical industry, there are three key variations to be discussed: EEA (specifically Norway), EFTA (specifically Switzerland) and World Trade Organisations (WTO) (Fig. 4).

#### EEA

The EEA, established in January 1994, currently includes Norway, Iceland and Liechtenstein. These countries implement EU legislation, such as free trade (except for agriculture and fisheries in most cases) and free movement, acknowledge EU administrative decisions, contribute to the EU to help level social and economic disparities across member states, and pay custom taxes and other administrative costs. However, they cannot vote in the European Parliament and have no says in its laws.

#### Norway.

The EEA model can be considered a poor deal for Norway since it is so similar to that of EU member states. However, Norway has retained some autonomy over its pharmaceutical sector. It has its own Medicines Agency (Statens legemiddelverk), which is a subsidiary to its national healthcare organisation. This is not so different to the UK where there is the NHS and the MHRA responsible for marketing medicines.

Although being part of the EEA means that Norway must adhere to EU regulations regarding marketing authorisations, its own Medicines Agency can influence the work of the EMA as EU member states can [50]. In addition, Norway has control over its own pricing and reimbursement, which is different for out- and in-patients, unlike for the rest of the EU [50].

There are therefore subtle differences in how Norway operates compared to that of EU member states, which could make it easier to sell this as a solution to the majority who voted for Brexit. In addition, considering that the UK’s existing framework is similar to Norway’s, it is feasible to envisage the UK transitioning to this model.

#### *Advantages of the EEA model for the UK* [51].

The EEA model would likely be the easiest option for the UK pharmaceutical industry, allowing for a transition to a legal framework only slightly different to the current model, whilst incentivising pharmaceutical companies to remain in the UK. An analysis of Norway suggests that the EEA model can succeed in maintaining and even attracting key players in the pharmaceutical industry; as of 2015, all major pharmaceutical companies were present in Norway with 9 having production facilities there [50].

Adopting an EEA model would therefore protect the status quo, allowing for continued organisation and efficiency between the UK and the rest of the EU in terms of R&D, clinical trials, manufacturing, marketing, distribution etc. This model would also enable pharmaceutical companies that are only based in the UK to benefit from the new reform starting in 2018 which will introduce a single EU portal for clinical trials. This will ensure a harmonised process for approval of clinical trials across the EU and enable participating nations to access and share clinical trial information on an EU database [52].

If the UK attains membership to the EEA, it effectively retains its status within the EU. This incentivises those EU pharmaceutical companies with registered offices or manufacturing sites in the UK, as well as those that conduct clinical trials in the UK, to continue their activities in much the same manner. Without this security net, these companies will have to demonstrate that their work complies with EU standards, which could prove time-consuming and expensive, possibly resulting in these companies leaving the UK.

This is especially relevant to those EU pharmaceutical companies that have no offices or manufacturing plants outside of the UK. Unless the UK joins the EEA, these companies will likely relocate to EU or EEA countries in the pursuit of operational ease and business security, as it will be disruptive and time-consuming to establish new legislative practices within a changing business environment to boot. Joining the EEA should therefore protect the UK pharmaceutical industry from the organisational chaos and economic detriment of pharmaceutical companies leaving the UK.

#### EFTA

The EFTA was formed in 1960 and, today, comprises Switzerland, Norway, Iceland and Liechtenstein. It allows for these four states to be incorporated into the EU’s single market. The EFTA is a prerequisite for joining the EEA.

As Switzerland is not also a member of the EEA (the Swiss rejected the idea in 1992), it has its own bilateral agreements with the EU, which took two years to finalise and cover all areas from trade to transport. The complexities of applying a similar model to the UK would therefore engender momentous negotiations.

#### Switzerland’s model.

‘Switzerland may guard its political and cultural independence fiercely, but its scientific sector has a strongly international flavour’ [53].

Switzerland is a rich country and that is partly thanks to its pharmaceutical industry, which is geared towards high value exports and supported by expert research. Switzerland is home to some of the world’s most successful pharmaceutical companies, such as Novartis and Roche, and noted for its scientific and academic institutions.

Despite not being an EU member state, Switzerland has also benefitted from EU FPs, such as Horizon 2020, which offer grants for research. The UK also has a strong reputation in the areas of science and research, and has received proportionately high funds through these programme (£67 billion alone through Horizon 2020). In fact, the UK receives more funding from the European Research Council than any other EU country and has priority access to scientific facilities across Europe, putting it at risk of losing a predicted £8.5 billion over the next four years [54].

Industry similarities and Switzerland’s economic success outside of the EU makes it unsurprising that many leave campaigners are championing a Swiss-inspired model as Brexit negotiations take shape. However, it seems highly unlikely that the EU will facilitate furthering these aspirations; in 2010, it was already referring to a relationship with Switzerland “which has become complex and unwieldy to manage and has clearly reached its limits” [55].

In addition, leave campaigners are motivated by what they view as Switzerland’s privileged position in terms of its unique relationship with the EU, yet many of them overlook the fact that the Swiss model aligns with many EU structures, laws and values. For example, in 1999, Switzerland accepted free movement of persons. Recently, Switzerland did indeed act to reinstate quotas on foreign workers. However, it was effectively punished by the EU which froze its Horizon 2020 grants and stalled its Erasmus + student mobility scheme [56]. This is a strong indication of the likelihood of failure if the UK attempts to negotiate entirely on its own terms.

#### WTO

Debate on this subject points to a third solution for the UK post-Brexit, that of the WTO, which is in fact the model that the UK will automatically revert to on exiting the EU [52]. This would be the most drastic option whereby the UK would abandon its European premise and use the established trade rules and norms of the WTO to forge bilateral trade agreements with the EU, resulting in a model similar to the rest of the world (that includes tariffs on trade with the EU, customs taxes etc.) [57].

This option could potentially offer the UK flexibility and the clean slate that leave campaigners rooted for, but it is the most ambiguous at this stage and would likely take many years to implement. For example, the UK could theoretically follow Canada which, after seven years of negotiations, signed the EU-Canada Comprehensive Economic and Trade Agreement (CETA) in 2013 and now profits from 98% tariff-free trade with the EU. Vicky Ford (Conservative MEP and Chair of the European Parliament Committee for the Internal Market and Consumer Protection) has stated that it is “much more important to look at the so called ‘non-tariff barriers’ which reflect the bureaucratic red tape faced by companies exporting into other markets and to recognise that the level of ease British companies currently have when selling into other EU markets is much, much greater than that which is now offered to Canada in CETA” [58]*.*

#### Final considerations

It should also be asked: is it really appropriate to compare the UK to Norway and Switzerland when demographically and economically these are very different nations? The former has a population of 5.1 million, the latter’s is 8.2 million. The UK has a population of 64.7 million and a GDP of $2.678 trillion compared to that of Norway and Switzerland at $512.6 billion and $685.4 billion respectively. The economic impact of having to be a ‘rule taker’ as opposed to a ‘rule maker’ on issues such as free movement is therefore likely to be far greater for the UK than for Norway or Switzerland [57].

There is also the historical and societal context. Switzerland and Norway never voted to leave the EU because they were never member states in the first place; Switzerland rejected joining the EU in 2001 with a vote of 76.8% and Norway likewise turned down the idea on smaller margins in referendums in 1972 and 1994.

### Moving forward

In 2011, the UK economy benefited by around £30 billion from pharmaceutical and chemical exports to the EU [59], which is just one of many figures serving to underpin the importance of investigating the impact of Brexit on this industry. The research conducted has yielded several policy recommendations and priorities based on their potential to maintain the UK’s attractiveness as a pharmaceutical hub post-Brexit.

#### Negotiate an ‘associated country’ status in the EU’s research funding programmes

This will guarantee access to the EU FPs and enable the UK to maintain its current dominance in the life sciences R&D sector. It will also sustain and encourage further collaborations between UK and European scientists, alleviating concerns over the uncertainty involved in working with UK-based partners. If the UK is to remain at the forefront of scientific innovation, it must work to preserve international collaborations.

#### Negotiate bilaterally favourable trade agreements for drugs and medical devices with the EU

The EU is an essential market for pharmaceutical companies in the UK. To prevent the exodus of pharmaceuticals companies currently based in the UK, the government must renegotiate trade conditions with the EU that are comparable to those pre-Brexit. This calls for a new streamlined customs system for UK-EU trade with low fee and administrative burden. This will also be important in preventing a sharp rise in the costs of drugs imported from the EU.

#### Mirroring the medicines regulatory approval process with the EMA, whilst retaining the MHRA’s capacity to intervene

This would bypass the need for pharmaceutical companies to seek separate product approvals in the UK. By opting to follow the EMA’s guidance, albeit with MHRA discretion for specific regulatory matters, the UK would incentivise pharmaceutical companies to remain in the country and prevent a delay in drugs reaching the UK market.

#### Assurance of free movement of high skilled professionals across UK-EU boarders

This will maintain the high skill level of the workforce in UK universities and the industry as a whole, whilst providing British nationals with the freedom to work, study and gain experience across the EU.

This option will appeal to multinational pharmaceutical companies who wish to quickly and easily relocate staff across international facilities. Free movement of professionals will therefore encourage foreign pharmaceutical companies to preserve their UK-based facilities. This will alleviate concerns regarding their EU staff members and their ability to attract and recruit the best in the field. Finally, such an agreement should encourage further foreign investment in the UK.

## Abbreviations

ABPI: Association of the British Pharmaceutical Industry
ATMP: Advanced Therapy Medicinal Product
CETA: Comprehensive Economic and Trade Agreement
CHMP: Committee for Medicinal Products for Human Use
CTD: Clinical Trials Directive
CTR: Clinical Trials Regulation
ECDC: European Centre for Disease Prevention and Control
EEA: European Economic Area
EFTA: European Free Trade Association
EU: European Union
FMD: Falsified Medicines Directive
FP: Framework Programme
GMP: Good Manufacturing Practice
GSK: GlaxoSmithKline
IPO: Initial Public Offering
MDD: Medical Devices Directive
MHRA: Medicine and Healthcare Products Regulatory Agency
NHS: National Health Service
NIHR: National Institute for Healthcare Research
PRAC: PV Risk Assessment Committee
SME: Small and Medium Enterprises
STEM: Sciences, Technology, Engineering and Mathematics
UUK: Universities UK
VMD: Veterinary Medicines Directive
